# Memory loss at sleep onset

**DOI:** 10.1093/texcom/tgac042

**Published:** 2022-10-29

**Authors:** Célia Lacaux, Thomas Andrillon, Isabelle Arnulf, Delphine Oudiette

**Affiliations:** Sorbonne Université, Institut du Cerveau - Paris Brain Institute - ICM, Mov'it team, Inserm, CNRS, 47-83 boulevard de l'Hôpital, Paris 75013, France; Sorbonne Université, Institut du Cerveau - Paris Brain Institute - ICM, Mov'it team, Inserm, CNRS, 47-83 boulevard de l'Hôpital, Paris 75013, France; Sorbonne Université, Institut du Cerveau - Paris Brain Institute - ICM, Mov'it team, Inserm, CNRS, 47-83 boulevard de l'Hôpital, Paris 75013, France; AP-HP, Hôpital Pitié-Salpêtrière, Service des Pathologies du Sommeil, National Reference Centre for Narcolepsy, 47-83 boulevard de l'Hôpital, Paris 75013, France; Sorbonne Université, Institut du Cerveau - Paris Brain Institute - ICM, Mov'it team, Inserm, CNRS, 47-83 boulevard de l'Hôpital, Paris 75013, France; AP-HP, Hôpital Pitié-Salpêtrière, Service des Pathologies du Sommeil, National Reference Centre for Narcolepsy, 47-83 boulevard de l'Hôpital, Paris 75013, France

**Keywords:** drowsiness, forgetting, memory, N1 sleep, sleep onset

## Abstract

Every night, we pass through a transitory zone at the borderland between wakefulness and sleep, named the first stage of nonrapid eye movement sleep (N1). N1 sleep is associated with increased hippocampal activity and dream-like experiences that incorporate recent wake materials, suggesting that it may be associated with memory processing. Here, we investigated the specific contribution of N1 sleep in the processing of memory traces. Participants were asked to learn the precise locations of 48 objects on a grid and were then tested on their memory for these items before and after a 30-min rest during which participants either stayed fully awake or transitioned toward N1 or deeper (N2) sleep. We showed that memory recall was lower (10% forgetting) after a resting period, including only N1 sleep compared to N2 sleep. Furthermore, the ratio of alpha/theta power (an electroencephalography marker of the transition toward sleep) correlated negatively with the forgetting rate when taking into account all sleepers (N1 and N2 groups combined), suggesting a physiological index for memory loss that transcends sleep stages. Our findings suggest that interrupting sleep onset at N1 may alter sleep-dependent memory consolidation and promote forgetting.

## Introduction

Numerous human studies have demonstrated that sleep improves or stabilizes memory in a variety of tasks, including perceptual, associative, spatial, motor, and emotional learning (Born et al. 2006; Diekelmann and Born 2010; Rasch and Born 2013; Paller et al. 2021). A growing body of research on animals supports the notion that this sleep-dependent memory consolidation is enabled by a hippocampo-neocortical dialog orchestrated by slow oscillations during NREM sleep (Maingret et al. 2016; Ólafsdóttir et al. 2018). Several studies suggest that sleep does not blindly consolidate all memories formed during the day but rather selectively consolidates those that are expected to be of future relevance (Saletin et al. 2011; Oudiette et al. 2013; Stickgold and Walker 2013; but, see Davidson et al. 2021 for a contrasted view on this topic). How the sleeping brain performs this “memory triage” remains mysterious (Stickgold and Walker 2013). One could imagine that the first sleep stage, known as NREM sleep stage 1 (N1 sleep), could contribute to this process by reviewing recent experiences and either deleting the memory traces deemed irrelevant (if the tagging process occurred presleep), or directly tagging the memory traces considered important for consolidation in subsequent sleep stages. However, because animal models used in sleep research lack an equivalent to the classically described NREM subdivision in humans (Lacroix et al. 2018), they cannot provide information about the role of each NREM stage, particularly N1, in memory. On the other hand, cognitive researchers have paid little attention to the N1 stage, possibly due to its fleeting nature, leaving us in the dark about its potential role in memory processing.

However, several factors suggest the involvement of sleep onset in memory processing. First, the hippocampus, a key brain region for memory consolidation, exhibits increased activity in the late N1 period compared to wakefulness (Picchioni et al. 2008). Second, N1 is associated with vivid, dream-like experiences (named “hypnagogia”) that often integrate recent daytime events (Stickgold et al. 2000; Wamsley, Perry, et al. 2010; Wamsley, Tucker, et al. 2010; Wamsley and Stickgold 2019). Moreover, memory-related experiences that are incorporated into sleep onset mentations are later found in the contents of NREM and REM sleep dreams within the same night of sleep (Wamsley and Stickgold 2019), and several studies have shown a positive correlation between dreaming about a task and subsequent memory performance (De Koninck et al. 1990; Wamsley, Tucker, et al. 2010; Plailly et al. 2019; Wamsley and Stickgold 2019). Combined, such findings would imply that the same memories are processed sequentially across the successive sleep stages (reminiscent of the “sequential hypothesis,” Giuditta 2014), supporting the aforementioned hypothesis that N1 acts as an initiating stage, tagging memories for consolidation during the subsequent NREM sleep (Stickgold 2009). This idea is substantiated by Stenstrom et al. (2012) who found that hypnagogic experiences connect fragments from distal memory sources that share semantic similarity, suggesting that a hippocampal-dependent integrative process (Shohamy and Wagner 2008) occurs during this stage (i.e. the blending of overlapping past events into an integrated memory representation). Recently, we confirmed the role of N1 in such a gist abstraction process (Lacaux et al. 2021). We found that spending, on average, 1 min in N1 tripled the chance of discovering a hidden regularity within mnesic traces (83%) compared to wakefulness (30%) or N2 sleep (14%).

However, beyond these findings, the literature on the sleep-onset period and memory remains sparse and inconclusive. One study showed that a 6-min nap was sufficient to improve word recall compared to a similar period of wakefulness (Lahl et al. 2008). By contrast, older studies reported evidence that sleep onset was associated with the retrograde amnesia of the materials encoded in the few minutes prior to sleep onset (Wyatt et al. 1994, 1997). However, in the aforementioned studies, the naps included both N1 and N2 sleep, thus preventing us from drawing a definitive conclusion about the respective contribution of each stage to the observed memory benefit.

Here, we aimed to better understand whether the twilight zone between wakefulness and sleep contributes to memory consolidation. To do so, we compared how a 30-min resting period that included either only wakefulness, only N1, or N1 + N2 sleep impacted the fate of memories that had been successfully encoded prior to the resting period.

## Materials and methods

### Participants

Fifty-two healthy volunteers (59.62% females, 24.83 ± 4.45 years; mean ± standard deviation [SD]) participated in the study. They were screened for exclusion criteria such as excessive daytime sleepiness as well as a history of sleep, neurological, or psychiatric disorders. To facilitate sleep onset, we asked participants to sleep about 30% less than usual during the night preceding the experiment and to avoid stimulants (e.g. coffee and tea) on the day of the experiment (which was conducted early in the afternoon). Subjects were paid 10€ per hour as compensation for their participation (plus a bonus based on their performance). All subjects provided their written informed consent prior to the onset of the study. The study protocol was approved by the local ethics committee (Comité de Protection des Personnes, Ile-de-France III, Paris, France).

### Task

**Fig. 1:**
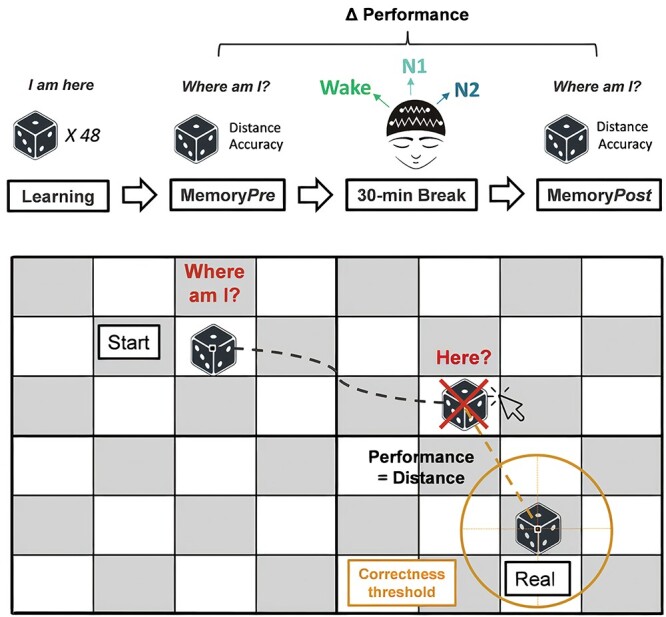
Experimental paradigm. (Top) Protocol timeline. Subjects first learnt the precise location of 48 pictures (e.g. a dice) which were presented within a grid. They were tested on their memory for these objects’ locations before and after a 30-min resting period during which they could sleep while being continuously monitored via polysomnography. They were regularly awakened (approx. every 6 min) by a sound to prevent them from sleeping too deeply and to probe their mental content. Based on their sleep–wake states during the resting period, participants were divided into 3 groups: Wake (*n* = 16), N1 sleep (*n* = 15), and N2 sleep (*n* = 21). (Bottom) Visuo-spatial memory test. Each object appeared at a wrong position (start) and the subjects had to drag it to its correct (real) location as precisely as possible. Here, the object would be considered as incorrect as the location provided by the subject is outside the circle’s perimeter, which represents the threshold for correctly placed objects (see Materials and methods for more details).

Subjects had to learn the precise location of 48 visual stimuli on an 8 × 6 squared grid background displayed on a 34.4-cm computer screen. Locations were randomly determined for each stimulus and each participant at the beginning of the session. Visual stimuli were pictures representing a variety of objects or animals and had a dimension of 156 × 156 pixels (corresponding to 4.13 × 4.13 cm). Subjects were instructed to reposition the pictures as precisely as possible in order to maximize their bonus monetary reward (maximum reward per picture = 10 cents). A black and white square indicated the center of the object and served as a reference point for the distance calculation. The “Psychtoolbox” (Brainard 1997; Kleiner et al. 2007) was used to program the task in Matlab R2018b. We computed two measures of accuracy for each object: (i) a continuous, precise measure consisting of the geometric distance between the position given by the participants and the correct position, and (ii) a binary correct versus incorrect measure. An object was considered correctly located if the participant placed it within 5.4 cm of its original location (corresponding to the diagonal of a square unit within the grid; see Fig. 1 for an illustration of the correct perimeter). This threshold was explicitly stated to participants and was the same as mentioned in Oudiette et al. (2013) and in Rudoy et al. (2009).

### Experimental design

Participants performed this spatial memory task (see Fig. 1 for details on the task) before and after a 30-min resting period while monitored with polysomnography. The protocol was divided into four main phases (summarized in Fig. 1).

#### Learning phase

Participants first went through a learning phase in which they had to memorize the precise location of 48 stimuli. First, the stimuli appeared 1 by 1 at the correct location, and each stimulus was repeated twice in a random order (passive viewing). The stimuli then appeared at a wrong location, and subjects had to drag them to the correct location using a computer mouse (active learning phase). They received feedback on their performance during this phase to ensure proper encoding. A picture was considered as “encoded” if the subject placed it correctly twice in a row; in that case, the picture was no longer presented during the learning phase (see the Task section for details on how accuracy is measured). The entire learning phase (passive viewing + active learning) was divided into 3 blocks of 16 stimuli, which were separated by a short break. It stopped when participants encoded the pictures’ positions well enough (i.e. 80% of correct stimuli). After completing the learning phase, participants were given a short, 3-min break before proceeding to the next phase (“Pre”).

#### Phase 2 (Pre)

Participants were tested on their memory for the 48 pictures (MemoryTest Pre). During the test, each object (1 at a time) appeared in a wrong position, and participants had to drag the object to its correct location using a computer mouse, just like in the learning phase, but without feedback this time.

#### Phase 3 (Break)

After the first memory test, participants were given a 30-min resting period. They lay on a bed in a dark room, eyes closed, with the instruction to rest and sleep if they wished. Every 6 min (plus a jitter randomly chosen from 0 to 30 s), we played an awakening sound to wake participants up. After each awakening, participants were instructed to speak aloud what was going through their mind (e.g. thoughts, images, reveries and dreams) before the alarm. This procedure aimed first at minimizing the probability that participants would fall into deeper sleep stages, thus maximizing the probability of obtaining resting periods with N1 as the only sleep stage. Second, it sought to assess the impact of spontaneous mental content on memory processing. According to this design, we later split participants into 3 distinct groups based on their sleep–wake state during the break (see demographic and sleep parameters in Table 1): a “Wake group” (subjects who stayed awake during the whole break; *n* = 16), a N1 group (subjects who reached N1 without any signs of deeper sleep stages; *n* = 15), and a N2 group (subjects who reached N2 stage; *n* = 21, of which 2 directly transitioned from wakefulness to N2 and therefore without any N1). Crucially, all experimental conditions were identical between these 3 groups.

**Table 1:** Demographic and sleep characteristics of each group.

Group	Wake	N1	N2	*P*-value	BF_01_
Demographic data					
Age, years	26.50 ± 4.50	24.10 ± 3.83	24.10 ± 4.68	0.20	2.04
Laterality (right-handed), %	100 (16/16)	93.33 (14/15)	85.71 (18/21)	0.27	8.32
Gender, women %	62.50 (10/16)	40 (6/15)	71.43 (15/21)	0.16	1.28
Educational level (0–7)	6.75 ± 0.45	6.67 ± 0.49	6.71 ± 0.46	0.88	6.09
Epworth score (0–24) Subjective sleep duration, h Subjective sleep onset latency, min Subjective sleep easiness (1–10)	7.50 ± 2.76 7.25 ± 0.93 19.80 ± 15.02 4.50 ± 2.58	8.0 ± 3.02 7.63 ± 0.61 18.13 ± 11.62 4.20 ± 2.18	7.90 ± 2.86 7.50 ± 1.12 16.45 ± 12.46 3.81 ± 2.14	0.87 0.41 0.79 0.66	6.04 4.15 3.24 4.89
Cofactors					
Distance Pre, cm	1.38 ± 0.38	1.48 ± 0.45	1.20 ± 0.31	0.17	1.02
Correct Pre, %	82.55 ± 10.72	83.47 ± 12.66	86.90 ± 8.96	0.48	3.52
PVT RT Pre, ms	292.79 ± 57.30	270.89 ± 18.98	275.21 ± 21.44	0.72	2.06
PVT RT Post, ms	274.66 ± 35.50	268.46 ± 24.49	280.36 ± 22.61	0.36	3.70
Sleep measures					
Wake duration, min	27.44 ± 1.05	23.26 ± 3.82	16.78 ± 5.40	**<0.001** ^**$**^	NA
N1 duration, min	0 ± 0	4.40 ± 3.75	3.78 ± 3.57	<0.001^*^	NA
N2 duration, min	0 ± 0	0 ± 0	7.22 ± 5.02	**<0.001** ^**#**^	NA
Sleep latency, min	NA	13.53 ± 7.06	9.43 ± 4.53	**<0.001**	NA

Measures are presented as the mean ± the SD or in percentage for proportions. *N*_Wake_ = 16, *N*_N1_ = 15, *N*_N2_ = 21. Educational level was scored according to the International Standard Classification of Education (Schneider 2013). Participants’ reported sleep duration, sleep onset latency, and sleep easiness (i.e. the ability to fall asleep in unfamiliar places such as in a train or hotel) were collected via questionnaires prior to the experiment. Data on Pre performance were computed only for objects that have been encoded during the training phase (see the Materials and methods section for more details). *P*-values are shown (ANOVA or Kruskal-Wallis Tests when appropriate: ordinal data or violations of normality assessed with the Shapiro–Wilk test, and Chi-squared Test for comparisons of proportions). When appropriate, post hoc comparisons have been computed with Tukey–Kramer correction for multiple comparisons, and we report significant differences between Wake and N1/N2 (^*^), N2 and Wake/N1 (#), and between all groups ($). Significant p-values are indicated in bold. Bayes factors, BF_01_, are reported in the event of nonsignificant differences.

#### Phase 4 (Post)

After the resting period, participants were tested again on the memory task (MemoryTest Post) using the same procedure as for the Pre test.

### Insight part

Of note, unbeknownst to the participants, the starting position of an object was not determined randomly but rather according to a hidden rule: It was always located on a fixed-length diagonal to the final/correct object’s location (mirror image, see Fig. 1 for an illustration). Once the objects’ locations were encoded (after the learning phase), participants were explicitly informed of a rule that would help them place any object within the grid (even new objects that they did not learn). We asked them regularly (before and after each phase) what the hidden rule was, and they had to tell us the solution they had in mind even if they knew that it was not the right one. This paradigm was originally designed to assess how the recent experiences are specifically transformed after N1 sleep (vs. wake rest and a nap containing N1 + deeper NREM sleep). Precisely, the task was developed to test at the same time whether N1 leads to changes in individual spatial memory traces (memory consolidation/forgetting) and to the sudden discovery of a hidden rule underlying all of these individual memories (insight).

Our initial hypothesis was that N1 would boost insight, which would be accompanied by the erasure of memory traces as they would no longer be needed (because knowing the rule would be sufficient to determine the position of any object). We were, however, unable to test the full hypothesis given that we did not have enough solvers (*n* = 11 across all groups, including only 7 solvers after the break), most likely because our newly designed task was too difficult. For the scope of this paper, we thus decided to focus on the memory part and to analyze only the participants who did not find the rule (*n* = 52). Nonetheless, the solvers’ performance on insight and memory is displayed in Supplementary Fig. S1. Here, memory performance was assessed using another implemented memory test in which the solvers could not use the rule (i.e. all the items were appearing in the screen’s center, making the rule impossible to use). Overall, we observed no between-group difference in the proportion of insight (Supplementary Fig. S1A). Memory performance across groups looked similar to that of nonsolvers (Supplementary Fig. S1B), even though the test for an absence of statistical difference between solvers and nonsolvers’ memory performance yielded inconclusive results (*P* = 0.29, BF_01_ = 1.53, Kruskal-Wallis), certainly due to a lack of statistical power.

### Vigilance assessment

To measure the potential differences in vigilance levels between groups, participants also performed a 3-min psychomotor vigilance test (PVT) at the beginning of phases 2 (Pre) and 4 (Post). The PVT consists of monitoring the appearance of a stimulus on a screen and responding to each appearance as fast as possible (Grant et al. 2017).

### Mental content

Each mental content reported during the resting period was labeled by the experimenters as either a dream or a thought, with each category further classified as task-related or not. Given that we were studying the wake-to-sleep transition, we opted for a more conservative definition of dream than the one commonly used (i.e. any mental content during sleep). Here, a mental report was only deemed a dream-like experience if it was “involuntary, spontaneous, perceptual, and bizarre (unusual, nonthought-like).”

### Sleep monitoring

Subjects were monitored with video-polysomnography for the entire duration of the experiment. The montage included 3 electroencephalography (EEG) channels (FP1, C3, and O1), electro-oculograms (EOG) with electrodes placed on the superior and inferior outer canthi of the eyes, chin electro-myogram (EMG), a microphone, and infrared video recordings. The impedances of electrodes were generally <5 kΩ. EEG signals were referenced to A2 (right mastoid) and were sampled at 250 Hz.

### Sleep scoring

EEG data were band-pass-filtered between 0.1 and 40 Hz and EOG derivations were band-pass-filtered between 0.3 and 15 Hz (2-pass Butterworth filter, fifth order). EMG signal was obtained with a local derivation placed on the chin, which was band-pass-filtered between 10 and 100 Hz (2-pass Butterworth filter, fifth order). Participants’ sleep–wake states during the resting period were scored offline by 2 experienced scorers according to the standard sleep scoring guidelines of the American Academy of Sleep Medicine (Berry et al. 2012). There was a high concordance between these 2 independent scorers (CL and DO, Kappa coefficient > 0.8), and the remaining disagreements were examined by a third expert scorer (SL). Specific grapho-elements (number of spindles and number of K-complexes) were also manually counted following the standard sleep scoring guidelines (Berry et al. 2012).

### E‌EG spectral analyses

A spectral decomposition of the preprocessed EEG signal was conducted on the entire resting period for the occipital O1 electrode. Epochs with an absolute amplitude >500 μV were discarded from this analysis. Welch’s method was used to estimate the log-transformed power spectral density for 2 frequency bands (alpha: 8–12 Hz and theta: 4–7 Hz) using a 6-s sliding-window with 50% overlap and a frequency resolution of 0.2 Hz. The power over each 6-s window was averaged for each 30-s epoch. Epochs with an absolute amplitude >150 μV were excluded from this analysis. Finally, the corresponding power spectra were averaged across the whole break duration and were log-transformed to ensure a pseudo-normal distribution (which explains why some power values are negative in Fig. 3A). A constant of 3 was added to the power values to guarantee that they were all >0, before calculating an alpha/theta ratio for each subject. Of note, the reported findings are similar for constant values ≥3. We also computed a normalized version of the alpha/theta ratio by *z*-scoring the individual values within each group (Wake, N1, and N2 sleep). The alpha/theta ratio was chosen as a canonical drowsiness index (theta power increases while alpha power decreases as we fall asleep) that has been used in numerous studies (see, e.g. Bareham et al. 2014; Comsa et al. 2019; Strauss et al. 2022).

### Statistical analyses

**Fig. 2:**
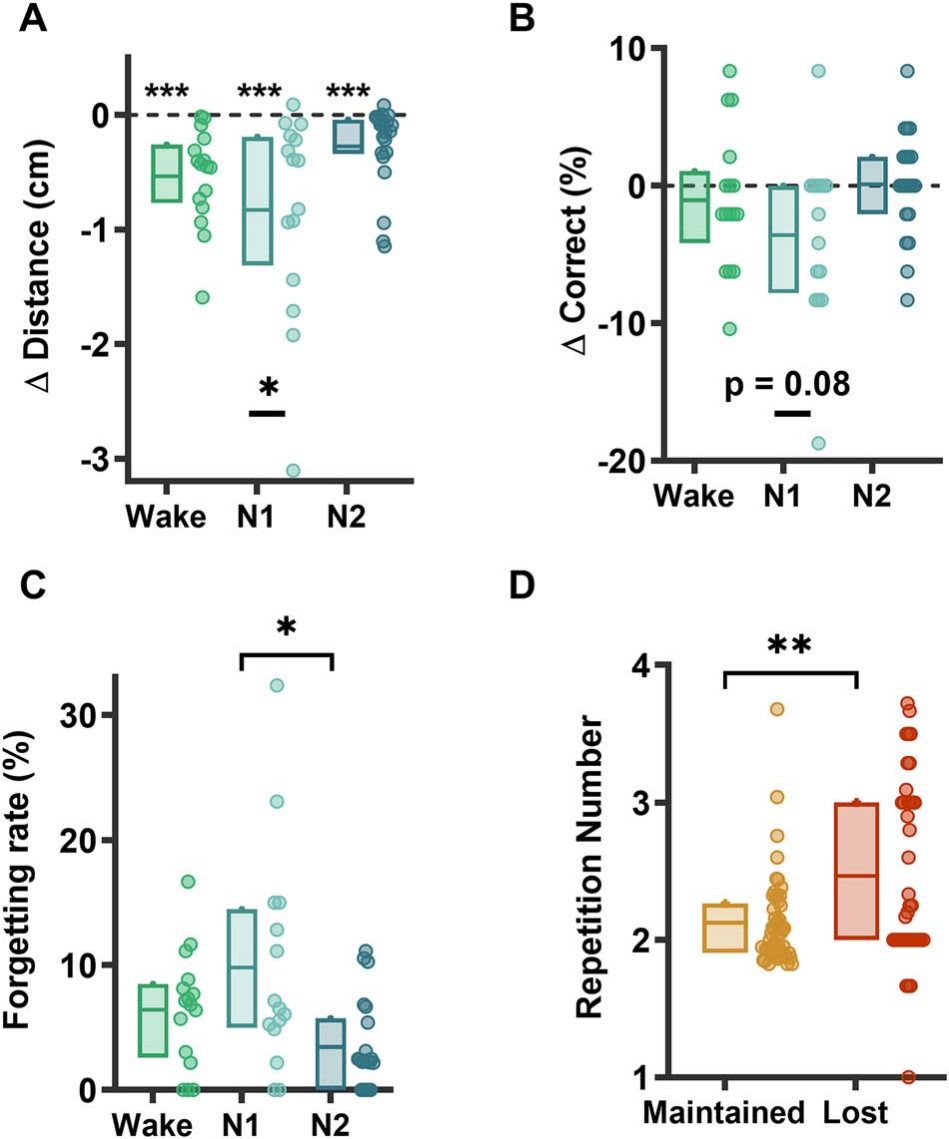
Memory loss at sleep onset. A) Delta (Pre-Post) distance and B) delta (Post-Pre) accuracy (percentage of correct objects) for each group (Wake, N1, and N2). Negative values thus indicate forgetting for both measures. The delta distance was computed on the items that were correct during the Pre test (items sufficiently encoded to potentially benefit from memory consolidation during the break). C) Forgetting rate for each group: $\Big(\mathrm{Nb}\ \mathrm{Lost}/\mathrm{Nb}\ \mathrm{Correct}\ \mathrm{Pre}\Big)\times 100$. D) Number of repetitions during the learning phase for the Maintained (correct in Pre and Post) and Lost items, all groups combined. For each box, horizontal lines represent the first, mean, and third quartiles. Individual data are also depicted by circles alongside their boxplot. Kruskal-Wallis tests were performed for comparisons between all 3 groups; when appropriate, post hoc comparisons with Tukey correction have been computed. One-sample Wilcoxon signed-rank was used for comparison with 0 level, and 2-sample Wilcoxon signed-rank was used for comparisons between 2 paired samples: Maintained and Lost. Stars are used alone to report the *P*-values obtained when comparing data to 0, stars underlined with a line for between-group differences, and stars above a square bracket for post hoc comparisons. ^*^^*^^*^  *P* < 0.001; ^*^^*^, *P* < 0.01; ^*^, *P* < 0.05.

As our aim was to investigate whether the sleep-dependent memory consolidation starts as early as N1, we restricted our behavioral analyses to items that were sufficiently encoded before sleep (apart from analyses on overall accuracy; Fig. 2B). Accordingly, we excluded data from pictures incorrectly recalled (i.e. pictures that were positioned above the distance threshold of 5.4 cm from the original location during the Pre test; mean = 7.40 ± 5.11 items excluded). Results on all items are provided in Supplementary Fig. S2. Fisher Tests were used to test relationships between categorical variables. Kruskal-Wallis Tests (or 1-Way ANOVAs for normally distributed data) were conducted to test the impact of the group (Wake, N1, or N2) on performance. When appropriate, additional post hoc comparisons with Tukey–Kramer correction for multiple comparisons were performed. Wilcoxon signed-rank Tests were used to compare 2 paired nonparametric variables and Mann–Whitney was used for independent samples. In the case of nonsignificant results, Bayesian statistics were computed using JASP (JASP Team 2019) with a prior distribution following a Cauchy distribution with a default scale rate of 0.707. A Bayes Factor BF_01_, typically >3 (i.e. the null hypothesis is 3 times more likely than the alternative hypothesis), provides supportive evidence for the null hypothesis.

Correlations between the alpha/theta ratio and memory performance were performed using Pearson’s correlation (and correlation plotting using the “gramm” toolbox; Morel 2018). The Cohen’s Κ test was used to evaluate interjudge agreement. All tests were 2-tailed, and a probability level of <0.05 was considered to be significant. All computations were performed using Matlab, version 2018b (The MathWorks Inc).

Finally, to estimate the respective influence of the subject groups (wake, N1, and N2) and alpha/theta ratios on the forgetting rate, we fitted a series of models with the forgetting rate as a predicted variable. The models were as follows:

Model 0: Forgetting-Rate ~ 1.

Model 1: Forgetting-Rate ~ 1 + Group.

Model 2: Forgetting-Rate ~ 1 + Group + Alpha/Theta.

Model 3: Forgetting-Rate ~ 1 + Group ^*^ Alpha/Theta.

These models were fitted using the “glm” function from the statistical package in R software. Model comparison was performed using the Anova function and the *F*-tests are reported in the Results section. Post hoc comparisons were performed on the winning model and by also using a *F*-test.

## Results

Participants performed a memory task before and after a 30-min break during which they were allowed to sleep (see Fig. 1 and Materials and methods for details on the experimental task and timeline). Throughout the break, subjects were regularly awakened (approximately, every 6 min) to prevent them from falling too deeply into sleep and to probe their mental content. Depending on their sleep–wake state during the resting period, participants were later subdivided into 3 groups (Wake, N1, and N2; see the Materials and methods for details).

Participants’ demographic and sleep parameters are provided in Table 1. The only difference that we observed between the Wake and N1 groups was the amount of time spent in N1 (respectively, 0 vs. 4.40 ± 3.75 min); subjects in the N2 group spent a similar amount of time in N1 than the N1 group (mean ± SD = 3.78 min ±3.57, *z* = 0.45, *P* = 0.65, Mann–Whitney U Test; BF_01_ = 2.86) plus an average of 7.22 (±5.02) min in N2.

### N1 sleep is associated with greater forgetting than N2 sleep

We found that the change in recall accuracy (geometric distance) after the resting period significantly differed as a function of the group (Wake, N1, and N2; Fig. 2A, χ^2^(2) = 6.82, *P* = 0.03, Kruskal-Wallis). The Pre-Post difference was <0 (i.e. subjects worsened their performance compared to Pre) for all groups (*P* < 0.001, Wilcoxon signed-rank tests), but this difference was larger for N1 subjects, who overall tended to place the objects further away from their correct location after the nap than before (mean Δ distance Pre-Post: Wake = −0.53 ± 0.42 cm, N1 = −0.83 ± 0.89 cm, N2 = −0.27 ± 0.36 cm, *P*-value between N1 and N2 = 0.055; post hoc comparisons with Tukey–Kramer correction for multiple comparisons). Results obtained with the binary (i.e. correct vs. incorrect) rather than the continuous estimate of recall, were concordant, and N1 subjects tended to have a lower overall accuracy than the Wake and N2 subjects (Fig. 2B, mean Wake = −1.04%, N1 = −3.61%, N2 = + 0.10%, *P* = 0.088, Kruskal-Wallis Test).

Importantly, the overall decrease in performance observed in the N1 group was not due to baseline differences in memory abilities, as all groups performed equally well at the Pre phase both in terms of average distance from the correct location (*P* = 0.17, BF_01_ = 1.02, Kruskal-Wallis) and number of correctly placed objects (*P* = 0.48, BF_01_ = 3.52, see Table 1). There was also no difference between groups in the number of trials required to complete the learning phase (mean Wake group = 108.06 ± 20.76, N1 = 108.73 ± 27.62, N2 = 100.38 ± 12.41 trials, *P* = 0.75, BF_01_ = 3.26, Kruskal-Wallis). Furthermore, control analyses indicate that the difference observed in the N1 group was not related to other confounding factors (e.g. sleep inertia), as all groups were not different in terms of sleepiness (Epworth sleepiness score), alertness (PVT score), or educational level (see Table 1). Of note, only 5 subjects out of 52 (9.62%) reported unambiguous task-related dreaming experiences, which does not allow statistical analyses on the role of mental content on memory performance. However, we collected a sufficient number of reported dreams in general or task-related thoughts to assess their impact on performance. There was no difference in the level of forgetting whether subjects reported dreaming experiences or not (mean forgetting rate in subjects without dream reports: 6.09% ± 4.54 vs. with: 6.29% ± 8.61; *P* = 0.23, BF_01_ = 2.07, Wilcoxon rank sum test), or if they thought about the task during the resting period (mean = 6.08 ± 5.94) or not (6.53 ± 6.90; *P* = 0.94, BF_01_ = 4.17, Wilcoxon rank sum test).

### An N1-related memory forgetting versus an N2-related memory consolidation?

We then investigated the link between memory performance and finer-grained sleep features. In the N2 group, we did not observe any correlation between the memory performance and N2 duration (*r* = 0.11, *P* = 0.62, Spearman correlation), or with the number of spindles (*r* = 0.13, *P* = 0.56, Spearman correlation) or the number of K-complexes (*r* = 0.12, *P* = 0.60, Spearman correlation). When combining all sleep subjects together (N1 and N2 groups), we noted a trend toward a positive correlation between the amount of time spent in N1 and memory forgetting (*r* = 0.31, *P* = 0.068, Spearman correlation). Last, we found no correlation between memory performance and other sleep parameters (independent of sleep stages), such as sleep onset latency (*r* = 0.04, *P* = 0.76, Spearman correlation), sleep duration (*r* = −0.00, *P* = 0.99, Spearman correlation), or the number of arousals (defined as any transition from N1/N2 to wake; *r* = 0.17, *P* = 0.33, Spearman correlation). Together, these results suggest that the difference between the N1 and N2 groups is driven by forgetting in N1 rather than memory consolidation in N2.

### Memory loss following N1 sleep concerns a subset of items

We then investigated whether this worsened performance was related to a general decrease in precision for all objects or if it was due to the forgetting of a subset of items. To do so, we classified the 48 objects into 4 categories: (i) Maintained (the remembered objects, correctly placed in both the Pre and Post phases), (ii) Gained (falsely placed at Pre, correctly placed at Post), (iii) Unlearnt (the forgotten objects, falsely placed in both the Pre and Post phases), and (iv) Lost (correctly placed at Pre, falsely placed at Post). Only the Lost objects category differed between groups (χ2(2) = 7.70; *P* = 0.02, Kruskal-Wallis Test; see Supplementary Fig. S3). Post hoc comparisons showed that there was a higher number of Lost objects (on average, 3.6 objects) following a resting period containing N1 sleep compared to one with N2 sleep (*P-*value between N1 and N2 = 0.02, between Wake and N1 = 0.66, and between Wake and N2 = 0.17; post hoc comparisons with Tukey correction). This corresponded to a 10% forgetting rate (Nb Lost/Nb Correct Pre)×100, Fig. 2C). Of note, this result also holds true when removing one outlier (defined as any data point that is 3 SD away from the mean) present in the N1 group (*P* = 0.04, Kruskal-Wallis Test).

### Labile memories are prone to forgetting

Additionally, we attempted to determine what these lost items had in common. Wyatt et al. (1994) reported a sleep onset-related amnesia of stimuli presented just prior to sleep onset (3 min before), with no deficit for stimuli presented earlier. Here, we did not observe a difference in the timing of the presentation of these items, respective to sleep onset, between those that remained correct and those that were lost during the active learning phase (3 possible blocks; mean presentation block: Lost = 1.92, Maintained = 2.06, *P* = 0.13, BF_01_ = 1.32) or during the Pre phase (48 stimuli; mean presentation order: Maintained = 23.74, Lost = 25.49, *P* = 0.18, BF_01_ = 2.98, Wilcoxon-signed rank). Instead, we found that the Lost objects were the ones that participants had originally the most difficulty encoding. Indeed, they were repeated more times during the learning phase before encoding than the Maintained objects (Fig. 2D; mean Lost = 2.46 repetitions, mean Maintained = 2.12; z = −2.74, *P* = 0.0061, Wilcoxon signed-rank Test). Of note, we did not observe between-group differences in the number of repetitions before the encoding of these Lost and Maintained objects (*P* = 0.41, BF_01_ = 3.63 and *P* = 0.75, BF_01_ = 4.31, respectively).

### Neurophysiological substrate of memory loss

**Fig. 3:**
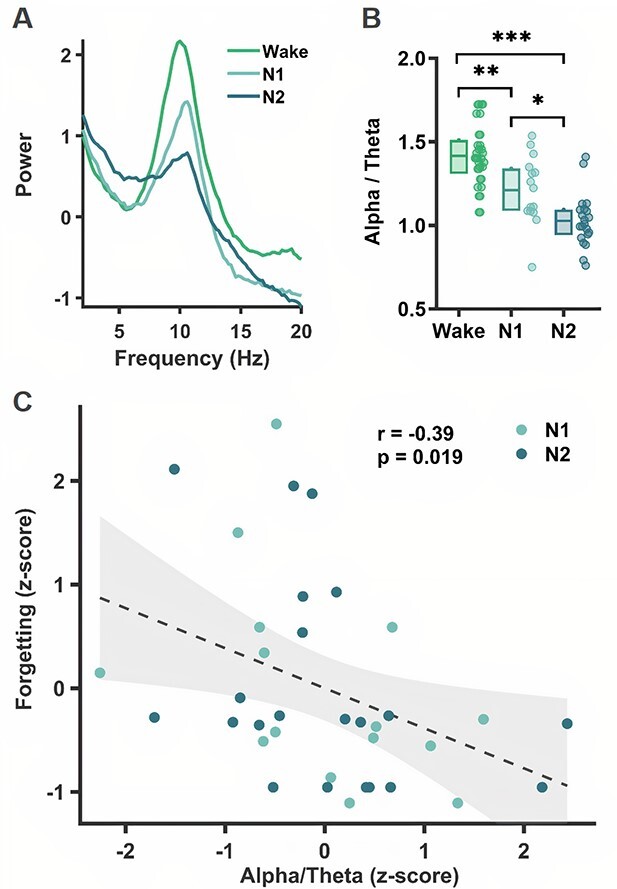
Neurophysiological substrate of memory loss. A) Average power spectrum and B) alpha/theta ratio over the occipital electrode during the Break for each group (Wake, N1, and N2). ^*^^*^^*^  *P* < 0.001; ^*^^*^, *P* < 0.01; ^*^, *P* < 0.05 (1-way ANOVA for comparisons between all 3 groups and Tukey–Kramer post hoc comparisons with correction for multiple comparisons). C) Pearson correlation between the forgetting rate (*z*-score) and the alpha/theta ratio (*z*-score) for all the subjects who slept (N1 and N2 groups). Both the raw individual data (circles) and a glm fit with a 95% confidence interval (line + shaded area) are plotted. Rho and *P*-values are displayed in the figure.

So far, we have categorized subjects according to standard sleep scoring methods. However, because it is based on discrete 30-s epochs, such classification misses subtle variations in the electrophysiological activity (Hori et al. 1994; Ogilvie et al. 2001; Hertig-Godeschalk et al. 2020) occurring at shorter timescales. To better understand the critical factors that may be associated with memory loss, we performed EEG spectral analyses over the entire break duration and explored how the power spectrum varied as a function of the group and memory performance (see Materials and methods). We first confirmed the expected between-groups difference in power spectral profiles (Fig. 3A) and alpha/theta ratios (a common marker of drowsyness, Fig. 3B), both of which showed a gradual increase in sleep depth between Wake, N1, and N2 subjects (alpha power and alpha/theta ratio differed between groups; mean alpha/theta ratio for Wake = 1.42, N1 = 1.21, N2 = 1.03; *F*(2, 49) = 21.12, *P* < 0.001, One-way ANOVA). Second and more importantly, a model comparison (see Materials and methods) showed that the model best explaining forgetting included both the group and the alpha/theta ratio (comparison of the full model with the model including only the group information: *F*(1, 48) = 4.54; *P* = 0.038; comparison of the full model with the model including only the alpha/theta ratio: *F*(2,48) = 7.70; *P* = 0.001). A model including an interaction component between Group and Alpha/Theta ratio was, however, not significantly better (*F*(2,46) = 1.45; *P* = 0.25). Considering this winning model, post hoc comparisons indicated that both the alpha/theta ratio and the subject group were predictive of the forgetting rate (*F*(1,48) = 4.54, *P* = 0.038; *F*(2,49) = 5.52, *P* = 0.0069 for Alpha/Theta ratio and Group, respectively). Alpha/Theta ratio was negatively associated with forgetting (*t* = −2.13; *P* = 0.038), and the N2 group showed less forgetting than both the Wake (*z*-ratio = 2.61; *P* = 0.025) and N1 (*z*-ratio = 3.89; *P* = 0.0003) groups, but there was no significant difference between the Wake and N1 groups (*z*-ratio = −0.61; *P* = 0.81).

To isolate the effect of the alpha/theta ratio, we normalized (*z*-scored) the alpha/theta ratios and forgetting rates within each group and quantified the correlation between these variables. We found a significant negative correlation between the alpha/theta ratio and the forgetting rate in all sleepers (including both the N1 and N2 groups; Fig. 3C; *r* = −0.39; *P* = 0.019, Pearson correlation; see Supplementary Fig. S4 for the correlation in each group separately), indicating that the more participants’ brain activity slowed down during the break (lower alpha/theta ratio), the more they forgot the pictures’ location. Of note, this correlation did not reach significance (only a trend) when Wake subjects were added (Supplementary Fig. S5; *r* = −0.25; *P* = 0.071, Pearson correlation). We also did not observe any significant correlation between the delta and sigma power and forgetting rate. Overall, these EEG results are consistent with our behavioral results since a low alpha/theta ratio (a good indicator of the N1 stage) was linked with a high forgetting rate. But they also extend the behavioral findings in two significant ways. First, they demonstrate that variations in the alpha/theta levels influence the extent of memory loss even within a single substage such as N1. Second, they provide a neurophysiological index for memory forgetting independent of sleep stages.

## Discussion

Countless studies on the relationship between sleep and memory have flourished in recent decades (Born et al. 2006; Diekelmann and Born 2010; Rasch and Born 2013). However, most of these studies focused on the role of the NREM sleep stages, N2 and N3, and, to a lesser extent, REM sleep, but left aside the specific impact of the first stage of NREM sleep (N1) on memory processing. Here, we devised an experimental paradigm, allowing us to isolate the specific role of N1 sleep in the fate of recently encoded memories. We found that a resting period, including only N1 sleep, was associated with a lower memory recall (~10% loss of previously encoded items) compared to a resting period, including both N1 and N2 sleep. Of note, sleep inertia upon awakening from N1 or N2 sleep is unlikely to explain these between-group differences in memory performance, as all groups had comparable levels of alertness at the beginning of the Post phase (see Table 1). Plus, we did not find a similar memory loss in N2 participants (who are more likely to experience sleep inertia upon awakening from N2 sleep than the subjects awakening from N1 sleep).

Using EEG spectral analysis, we also found a negative correlation between the ratio of alpha/theta power (a marker of alertness) and the forgetting rate. This means that, for both N1 and N2 subjects, a higher level of drowsiness (lower alpha/theta ratio) was associated with a higher forgetting rate. This result could seem at odds with the increase in forgetting observed in the N1 group compared to the N2 group. However, this ratio was calculated over the entire break and therefore does not specifically reflect what happened in N2 sleep but rather an overall level of drowsiness. Furthermore, one could hypothesize that opposite neural mechanisms specific to N2 (e.g. sleep spindles, K-complexes, and slow-waves, which are absent in N1 sleep), which are not captured by the alpha/theta ratio, would counteract the N1-forgetting effect in the N2 group. This interpretation, however, is inconsistent with the current analysis, which shows no correlation between the hallmarks of N2 and memory performance. Nonetheless, the average duration of N2 was short (only 4 min on average) compared to traditional sleep studies showing a beneficial effect of N2 on memory consolidation (Marshall et al. 2006; Mednick et al. 2013; Ngo et al. 2013; Cairney et al. 2018). We might thus have a heterogeneous group in terms of N2 “sleep depth,” making it more difficult to unravel the putative beneficial effect of some N2 features on memory.

Overall, our findings suggest that N1 sleep specifically yields to the forgetting of recently encoded memories, particularly the ones that were encoded with the greatest difficulty.

What role does N1 sleep play in sleep-related memory processing? We see three possibilities. First, in accordance with the synaptic downscaling hypothesis (Tononi and Cirelli 2006, 2014), N1 sleep may be involved in the active suppression of items with a weaker synaptic weight (the Lost objects in our study). Such erasure of information could be necessary to make room in the synaptic network for subsequent memory consolidation in N2 sleep. Besides, forgetting is viewed as an essential function of sleep for efficient learning, complementary to memory consolidation (Hardt et al. 2013; Feld and Born 2017; Poe 2017). A few studies indicate that sleep actively contributes to forgetting by preferentially consolidating some information and pruning out others (Payne et al. 2008). Additionally, Feld et al. (2016) showed a sleep-dependent forgetting effect when the amount of encoded information was large. The authors hypothesized that large-scale data encoding results in overlapping hippocampal representations (Feld et al. 2016). During the following sleep, the gist (overlap) would then be preferentially consolidated, and the memory traces would be pruned out following global synaptic renormalization. This process could occur during N1 sleep and account for the observed forgetting of a few items.

An alternative hypothesis would be that N1 sleep instead initiates memory reprocessing by tagging, through reactivation, the memory traces most in need of being reinforced (the weaker ones) by subsequent sleep stages. In that case, by preventing deeper sleep from supplanting N1 sleep, we would have aborted this process, resulting in the forgetting of these peculiar items (potentially by destabilizing those reactivated memory traces; Bonin and Koninck 2015). Further studies would be needed to disentangle between these hypotheses, for example, by using multiple short naps of different nature with memory tests performed between them (e.g. testing whether a second nap, including N2 sleep rescues memories that had been forgotten after a first nap including only N1 sleep). Last, recent research indicates that arousals and sleep disturbance (in their case, caused by the Targeted Memory Reactivation method) are associated with memory deterioration (Göldi and Rasch 2019; Whitmore et al. 2022). Thus, the forgetting effect observed in N1 may be the result of the multiple awakening procedure that we used. Related to this point, one intriguing question for further studies will be to assess whether N1 only impacts memory when present at the beginning of a nap/night or if it also plays a role when occurring later (e.g. after an awakening from sleep).

Whichever hypothesis turns out to be correct, the present findings indicate that some kind of memory processing might be occurring at sleep onset and highlight two different patterns between two seemingly proximal sleep stages, N1 and N2 sleep. Interestingly, these findings parallel those we recently observed about creativity (Lacaux et al. 2021). Indeed, we discovered that N1 sleep was associated with a boost in insight (i.e. the sudden discovery of a hidden regularity in the task), but this benefit vanished if subjects reached N2 sleep. This analogy suggests that (i) memory and creativity may be intertwined and (ii) it is important to consider the N1 and N2 sleep stages separately in future studies. Unfortunately, we did not have enough subjects who reported unambiguous task-related dreams to evaluate their impact on subsequent performance. Whether task-related dreaming directly impacts memory performance (as implied by some studies; Wamsley, Tucker, et al. 2010; Wamsley and Stickgold 2019), or is merely an epiphenomenal reflection of ongoing memory, reprocessing remains an open question.

Our study has several limitations. First, we assigned participants to a group ad hoc based on their sleeping pattern during the break. Confounding variables (e.g. sleepiness and discomfort) may have influenced our subjects’ wake/sleep trajectory. Unfortunately, this is a problem inherent to sleep research, given that we cannot force participants to fall asleep or remain awake (without adding additional biases such as stress). We attempted to minimize these putative confounding variables by using the exact same experimental procedure for each subject (same level of sleep deprivation, armchair, EEG setup, and level of darkness). While hidden variables may have influenced participants’ sleep/wake states, we believe that this does not alter our main conclusion that following the N1 trajectory (whatever the reason) is associated with greater forgetting. Second, while our results provide indirect evidence of an association between N1 and memory reprocessing, further studies will be necessary to test whether this link is causal or reflects other, hidden processes. Furthermore, some aspects of our experimental design might have influenced memory performance, such as offering a monetary reward depending on one’s performance or the fact that our task was originally designed to test two cognitive components (memory and insight). However, these putative confounding factors were similar in all groups. Finally, while we observed a difference in memory performance between the N1 and N2 groups, we did not find any differences between the wake group and any of the sleep groups. Thus, our results could be interpreted in two ways: either they add evidence to the well-known memory consolidation effect associated with N2, or they suggest that N1 promotes forgetting. More research is needed to distinguish between these two interpretations, but several lines of reasoning point to the latter. First, we observed no difference between the Wake and N2 groups, which is in contrast with the classical NREM sleep-related memory consolidation effect. Additionally, we found no correlation between N2 features (e.g. number of spindles or K-complexes) and memory performance, whereas some N1 markers (N1 duration and alpha/theta ratio) were correlated with memory performance (for all sleeping subjects, including the N2 group).

## Conclusion

In conclusion, by using a novel design that separates N1 from N2 sleep, we discovered that memory processing also takes place during N1 sleep, a stage that appears to promote memory forgetting. Interestingly, this function was originally hypothesized to be associated with another sleep stage, REM sleep (Crick and Mitchison 1983), a theory that has recently received empirical support (Li et al. 2017; Izawa et al. 2019). These latest studies provide a putative mechanism underlying forgetting via the pruning of certain synapses, a mechanism that could thus occur as early as during N1 sleep. We hope that our work will launch further investigations to corroborate such results and to better understand the mechanisms at work during N1 sleep and its role in determining the fate of our memories.

## Supplementary Material

Lacaux_Supplementary_tgac042

## Data Availability

All the relevant data are available upon reasonable request. Inquiries should be directed to the corresponding author.
