# Supplementary material for: Memory loss at sleep onset
Source: Cereb Cortex Commun. 2022 Oct 29;3(4):tgac042. doi: 10.1093/texcom/tgac042 (PMC9677600; doi:10.1093/texcom/tgac042)
Supplement: Lacaux_Supplementary_tgac042 [file lacaux_supplementary_tgac042.docx]

**SUPPLEMENTARY FIGURES**

**
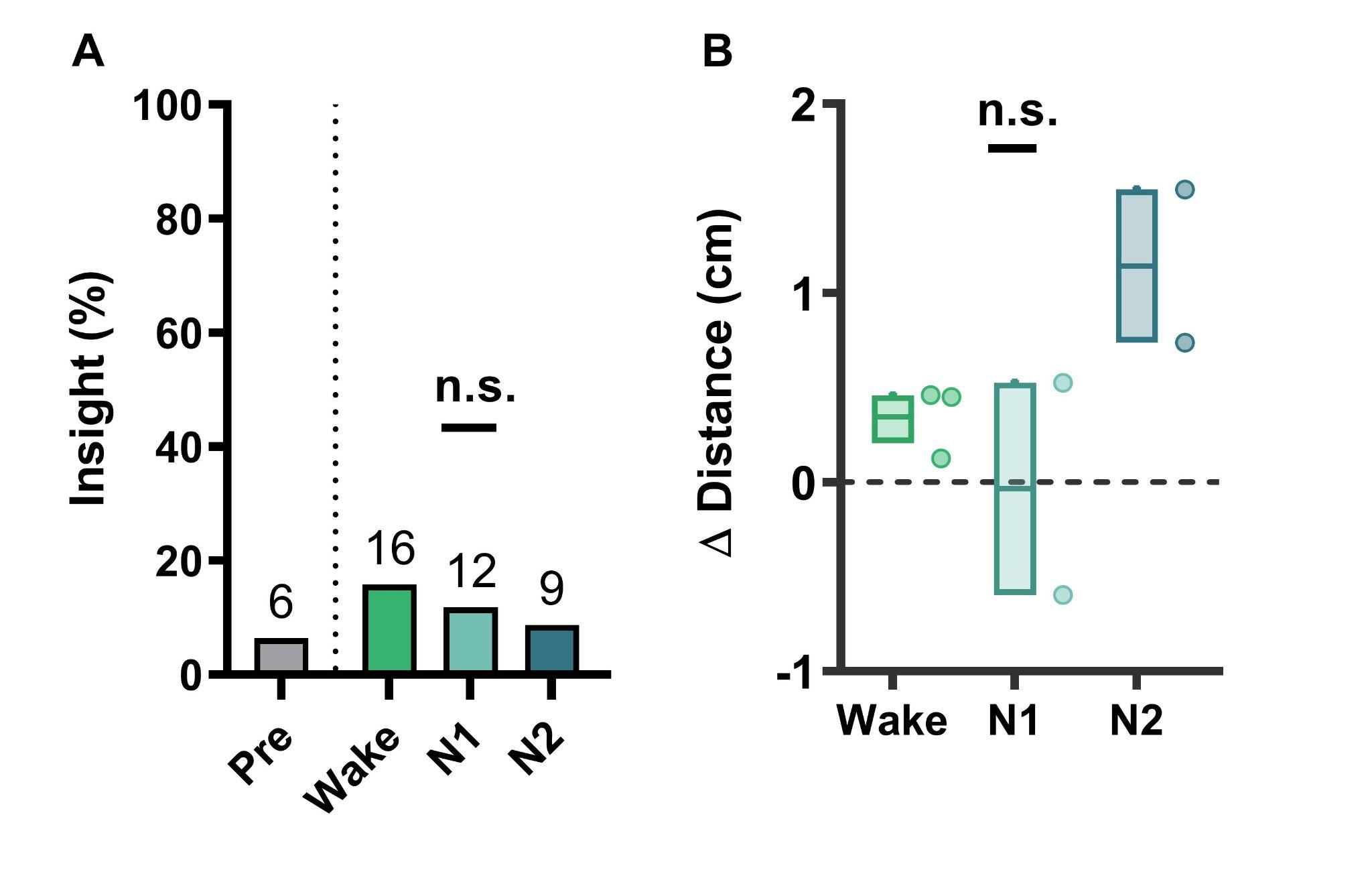
**

***Figure S1 - Performance in solvers. (A)*** *Percentage of insight (i.e., discovery of the hidden rule) before and after the break (Wake, N1, and N2 defined by participants’ sleep/wake state during the break; number of solvers Pre = 4, number of solvers Post = 7, including 3 in the Wake group, 2 in the N1 group, and 2 in the N2 group).* ***(B)*** *Delta (pre-post) distance in the visuo-spatial memory task for each group (Wake, N1 and N2). n.s., non-significant between-group differences.*

**
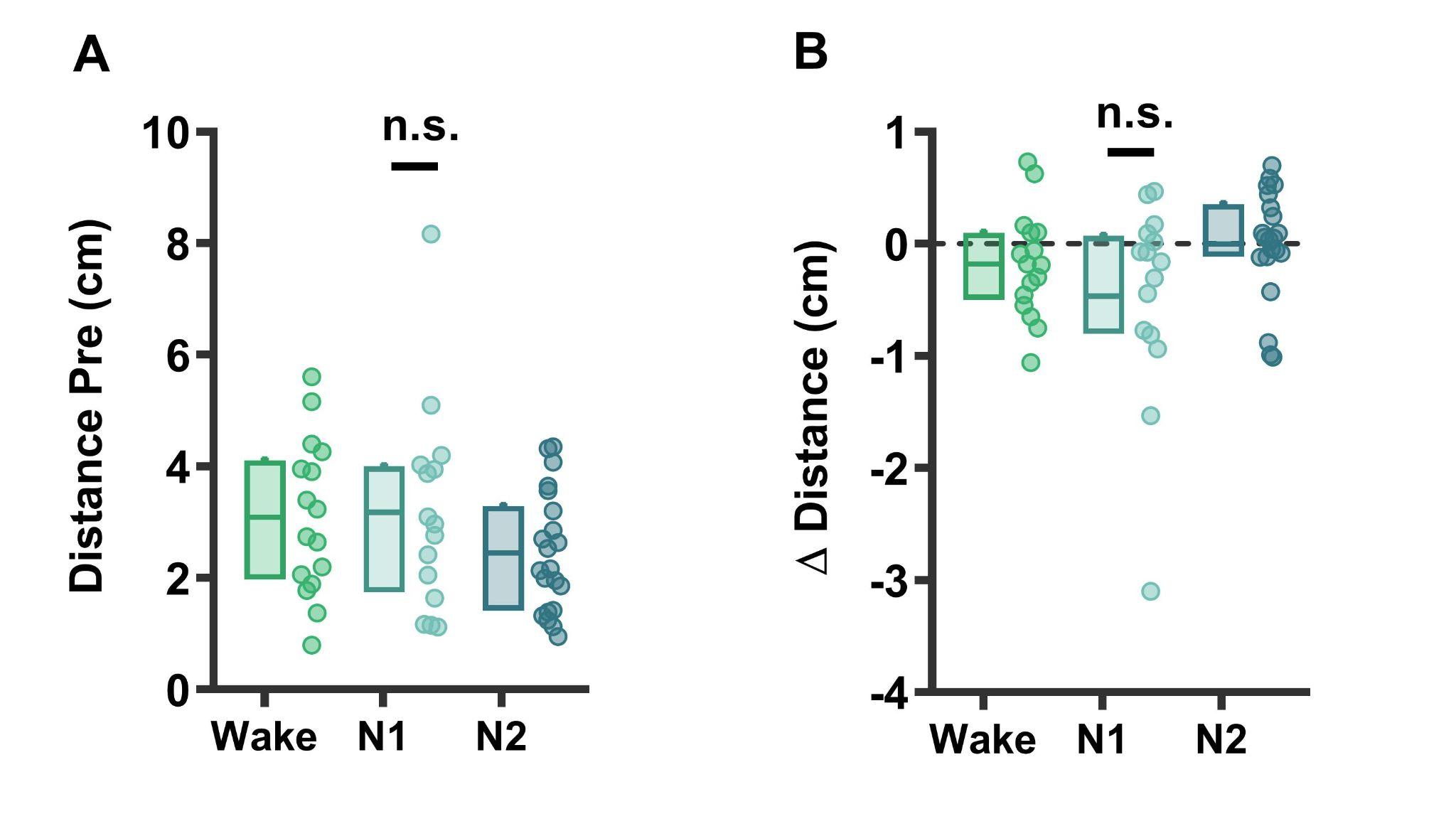
**

***Figure S2 - General performance. (A)*** *Distance from correct location in the Pre phase and* ***(B)*** *delta (pre-post) distance on all items (including the unlearnt) for each group (Wake, N1 and N2). n.s., non-significant between-group differences (Kruskal-Wallis).*

*
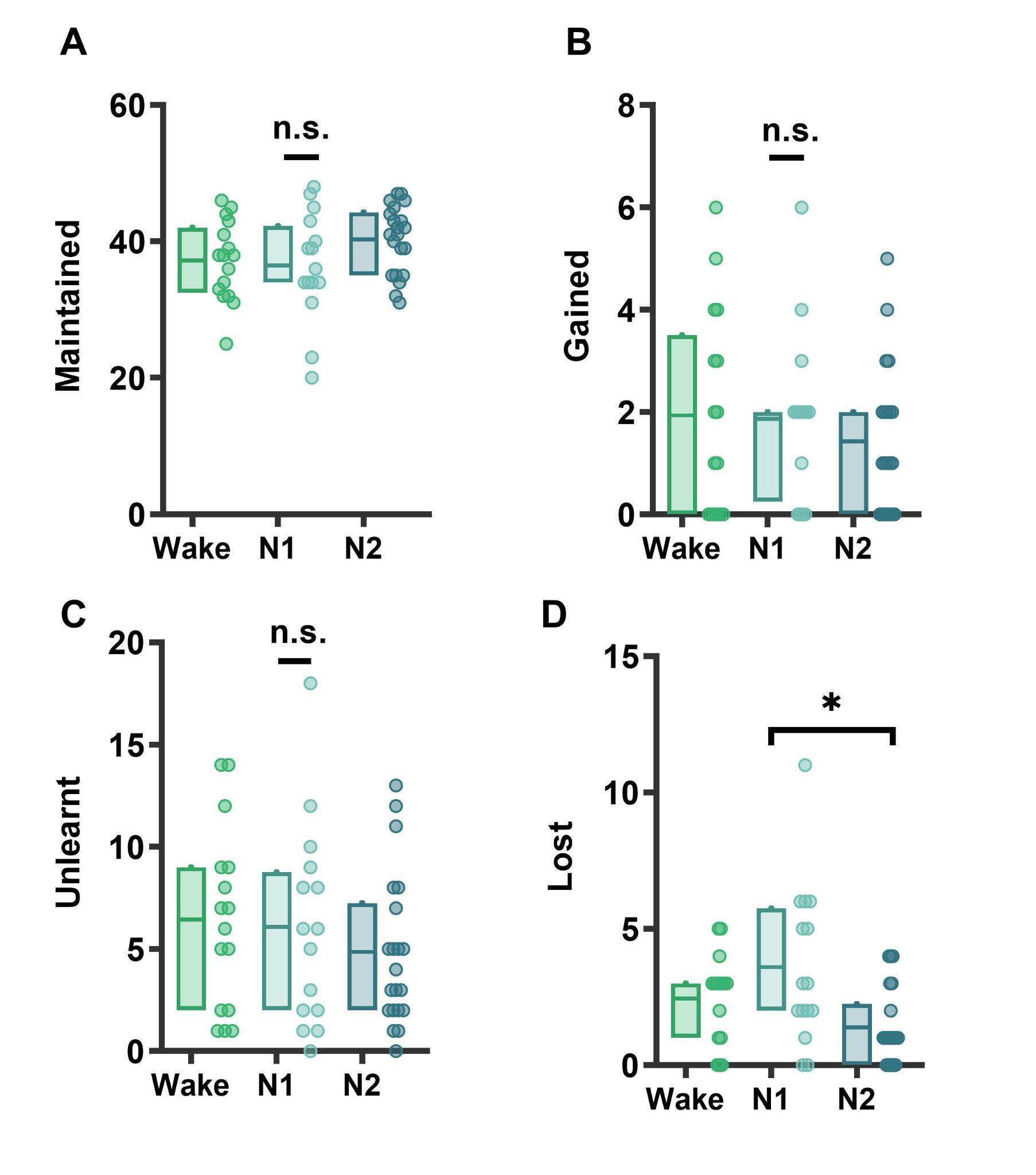
*

***Figure S3 - Repartition of the objects.*** *The number of objects in each of the 4 defined categories for all groups:* ***(A)*** *Maintained: objects correctly located in both the Pre and Post phases,* ***(B****) Gained: objects falsely located in the Pre phase but correctly located in the Post phase,* ***(C)*** *Unlearnt: false in both the Pre and Post phases, and* ***(D)*** *Lost: correctly located in Pre but falsely located in Post.* **p<0.05; n.s., non-significant between-group differences (Kruskal-Wallis).*

*
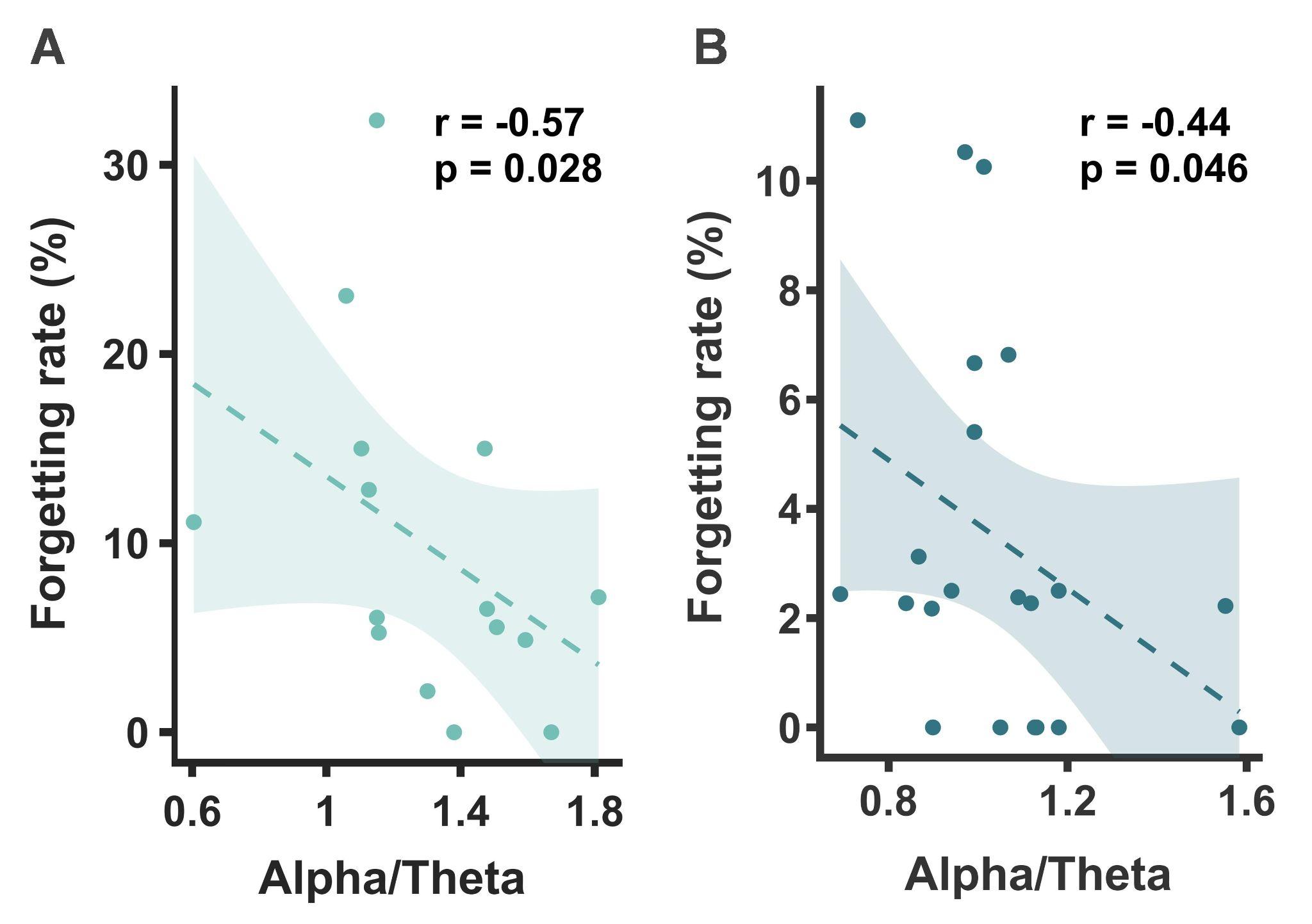
*

***Figure S4 - A neurophysiological marker of forgetting.*** *Spearman correlations between the forgetting rate and the alpha/theta ratio for the N1* ***(A)*** *and the N2* ***(B)*** *groups. Both the raw individual data (circles) and a glm fit with a 95% confidence interval (line+shaded area) are plotted. Rho and p-values are displayed in the figure.*

*
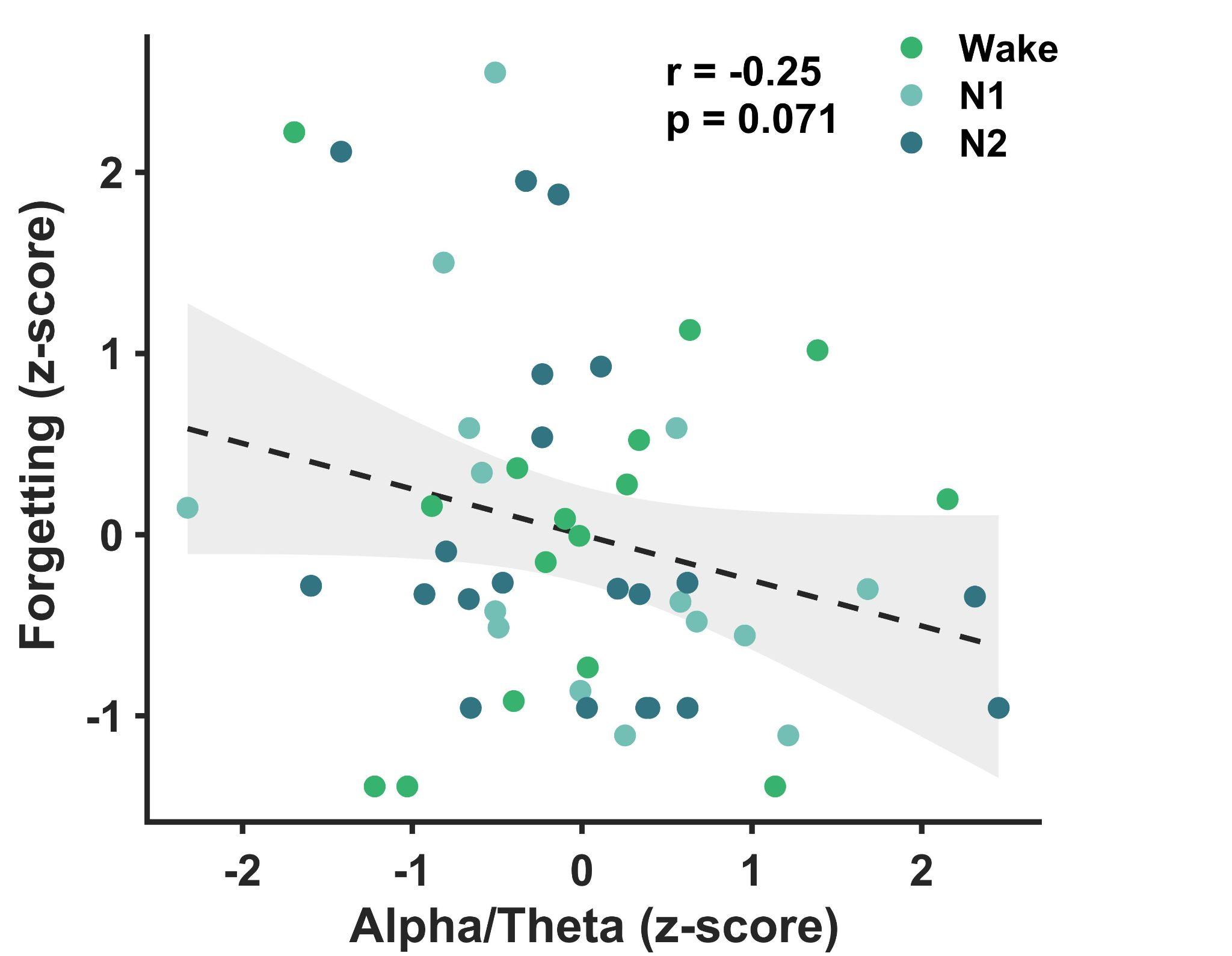
*

***Figure S5 - Pearson correlation between forgetting rate (z-score) and alpha/theta ratio (z-score)*** *for all subjects (Wake, N1 and N2 groups). Both the raw individual data (circles) and a glm fit with a 95% confidence interval (line+shaded area) are plotted. Rho and p-values are displayed in the figure.*
